# Single-Cell RNA Sequencing Unravels Distinct Tumor Microenvironment of Different Components of Lung Adenocarcinoma Featured as Mixed Ground-Glass Opacity

**DOI:** 10.3389/fimmu.2022.903513

**Published:** 2022-07-06

**Authors:** Yu He, Fenglei Yu, Yi Tian, Qikang Hu, Bin Wang, Li Wang, Yan Hu, Yongguang Tao, Xiaofeng Chen, Muyun Peng

**Affiliations:** ^1^ Department of Thoracic Surgery, The Second Xiangya Hospital of Central South University, Changsha, China; ^2^ Department of Thoracic Surgery, Beijing Hospital, National Center of Gerontology, Institute of Geriatric Medicine, Chinese Academy of Medical Sciences, Beijing, China; ^3^ Department of Anaesthesia, The Second Xiangya Hospital of Central South University, Changsha, China

**Keywords:** ground-glass opacity, non-small cell lung cancer, intra-tumor heterogeneity, single-cell RNA sequencing, macrophage

## Abstract

Lung adenocarcinoma featured as mixed ground-glass opacity (mGGO) doubled its volume half of the time in comparison with that featured as pure ground-glass opacity (pGGO). The mechanisms underlying the heterogeneous appearance of mGGO remain elusive. In this study, we macro-dissected the solid (S) components and ground-glass (GG) components of mGGO and performed single-cell sequencing analyses of six paired components from three mGGO patients. A total of 19,391 single-cell profiles were taken into analysis, and the data of each patient were analyzed independently to obtain a common alteration. Cancer cells and macrophages were the dominant cell types in the S and GG components, respectively. Cancer cells in the S components, which showed relatively malignant phenotypes, were likely to originate from both the GG and S components and monitor the surrounding tumor microenvironment (TME) through an intricate cell interaction network. *SPP1* ^hi^ macrophages were enriched in the S components and showed increased activity of chemoattraction, while macrophages in the GG components displayed an active antimicrobial process with a higher stress-induced state. In addition, the CD47–SIRPA axis was demonstrated to be critical in the maintenance of the GG components. Taken together, our study unraveled the alterations of cell components and transcriptomic features between different components in mGGOs.

## Introduction

For early-stage lung adenocarcinoma (LUAD), one of the radiological features could be pulmonary ground-glass (GG) opacities (GGOs), which are usually managed clinically based on their sizes, locations, growth rates, and percentages of the solid (S) components (1–3). Based on whether existing the S components, GGOs are usually classified as pure GGOs (pGGOs), which are entirely composed of the GG components, and mixed GGOs (mGGOs), containing both the GG and S components. According to epidemiological investigations, it is now generally accepted that lung cancers featured as mGGOs imply a higher likelihood of disease progression in comparison with those featured as pGGOs (4, 5). Several clinical trials have shown that the increasing ratio of the S components leads to poor prognosis (6, 7). Furthermore, a recent study found limited response of GGO to the PD-1/PD-L1 therapy, suggesting different immune microenvironments between GGO and advanced lung cancer (8). Therefore, the mechanism underlying the specific features of GGO should be studied, and novel strategies to manage the disease should be proposed.

Radiological features of mGGOs have been largely investigated, as well as common genetic alterations of GGOs were also characterized using bulk sequencing. The most frequent mutation identified in GGOs in Asia is *EGFR* mutations, while mutations in common lung cancer driver genes such as *TP53*, *ALK*, and *KRAS* are only sporadically found in GGOs (9–13). This indicates that different molecular features could be found in GGOs. However, limited studies have been performed in understanding the development of GGOs, especially interpreted distinct growth patterns among the S and GG components within mGGOs. One of the reasons is that malignant cells in GGOs are hard to investigate by using traditional bulk sequencing techniques due to their limited fractions in the lesions. Furthermore, the crosstalk between malignant cells and immune cells is also essential in the progression of neoplasia (14). To decipher intricate single-cell profiles, single-cell RNA sequencing (scRNA-seq) was introduced.

In recent years, the number of scRNA-seq research on GGOs has exploded. Lu et al. found higher heterogeneity in clonal architectures of solid LUAD tumors compared to pGGO by scRNA-seq (15). Another study also demonstrated the differences among solid LUAD tumors, mGGOs, and normal lung tissues and depicted the distribution of the various cell types in mGGOs (16). These prior studies have provided valuable information to outline the overall molecular characteristics of GGOs. However, the molecular-level differences between the S and GG components, and whether there is a developmental relationship between the two types of components remain unclear.

From real-world clinical observations, the different proportions between the S and GG components, or the consolidation-to-tumor ratio (CTR) in mGGOs, implied distinct fate of disease progression (17, 18). From a pathological point of view, the S portion of mGGOs exhibits infiltrative characteristics and thus is considered more malignant than the GG components. However, the factors driving these infiltrative characteristics are currently not well defined. In this study, we focused on the intra-nodule heterogeneity of mGGO nodules and attempted to identify the key molecular mechanisms underlying the transformation from early pre-infiltrative lesions to minimally invasive adenocarcinoma (MIA) or invasive adenocarcinoma. In this regard, we performed scRNA-seq on paired GG and S component tissues from three patients with pulmonary mGGO nodules of similar sizes in which the GG and S portions were clearly demarcated and investigated potential mechanisms underlying the progression of mGGOs.

In this study, we depicted distinct cell atlas and transcriptomic features of different components in mGGOs. The dominant cell types within the lesions, cancer cells, and macrophages were extensively discussed. Coupled non-negative matrix factorization (cNMF) was used to decipher common and unique changes in cancer cells and macrophages in different components. Furthermore, the evolution of cancer cells was analyzed using two different ways of trajectory analysis. Furthermore, we defined 4 component-enriched macrophage subsets from 12 macrophage subclusters in mGGO samples. Finally, alterations of comprehensive cell networks were described, and region-specific intercellular interactions were explored. To eliminate inter-patient heterogeneity, most of the results were obtained after intersecting data from separate analyses in a patient-independent way. Taken together, our study explored a new strategy to investigate human tumor samples and provided a comprehensive transcriptomic overview of the GG and S components of mGGOs for the first time.

## Material and Methods

### Patients and Tissue Samples

Surgical tissue samples were collected from three patients who underwent surgical resections of mGGOs at the Second Xiangya Hospital of Central South Hospital from October 2019 to April 2020. The inclusion criteria were as follows: 1) the radiological manifestation was mGGO, 2) the solid area was relatively flat and clear demarcated from the GG area under high-resolution CT (HRCT), 3) patients were disease free except for lung nodules, 4) patients had not received any antitumor therapy before the surgery, and 5) pathological examination suggested invasive LUAD. Detailed clinical information is shown in **Supplementary Table 1**. This study was approved by the Ethics Committee Board of the Second Xiangya Hospital of Central South Hospital (2020084). Written informed consent was obtained from all participants included in this study.

### Tissue Dissociation and Single-Cell RNA Sequencing Sample Preparation

Paired patient tissue samples of the S and GG components from the same mGGOs were collected during surgeries. An incision along the largest cross-section of the mGGO nodule was performed, and naked-eye identification and CT were used to map the focal areas of the S and GG components. Dissected areas were identified by at least two first-line thoracic surgeons based on CT images and tissue appearance. The GG areas usually appeared grayish white, while the solid areas usually appear dark gray or waxy white, with visible blood vessels and fine bronchial perforations. To avoid mixtures between different focal areas, the GG components distal to the nodule were taken, and it was ensured that the dissected solid focal areas were smaller than their actual sizes.

The samples were stored at 4°C in the GEXSCOPE™ Tissue Preservation Solution (Singleron Biotechnologies, Nanjing, China) and processed on ice within 72 h. After samples were washed 3 times with Hanks’ Balanced Salt Solution (HBSS) and minced into 1–2-mm-thick pieces, 2 ml of GEXSCOPE™ Tissue Dissociation Solution (Singleron Biotechnologies, Nanjing, China) was added to each sample and allowed for digestion at 37°C for 15 min to dissociate the tissue into single cells. Then, the samples were filtrated using 40-μm sterile strainers, followed by centrifugation at 1,000 rpm for 5 min. After the centrifugation, the precipitation was resuspended in 1 ml of phosphate-buffered saline (PBS; HyClone, Marlborough, MA, USA). Subsequently, 2 ml of GEXSCOPE™ Red Blood Cell Lysis Buffer (Singleron Biotechnologies, Nanjing, China) was added to the cell suspension and incubated at 25°C for 10 min to remove red blood cells. After that, the mixture was centrifuged at 500 × *g* for 5 min, and the precipitation was resuspended in PBS. Finally, trypan blue (Millipore Sigma, Merck KGaA, Darmstadt, Germany) was used to stain the samples, and the cell viability was evaluated under a phase-contrast light microscope (Nikon, Tokyo, Japan).

### Tissue Density Calculation

Tissue density was obtained by the equation below after calculating the wet weight of dissociated tissue (including samples excluded due to non-compliance with the requirements such as pathological typing):

Tissue density = tissue weight (mg)/[number of dissociated cells/10 (4)]

This indicated the weight of tissue occupied per 10,000 cells.

### Single-Cell RNA Sequencing and the Primary Analysis of Sequencing Data

GEXSCOPE™ Single Cell RNA Library Kit Tissue (Singleron Biotechnologies, Nanjing, China) was used to barcode single cells, capture mRNA from isolated single cells, and generate cDNA libraries for scRNA-seq. Then, individual libraries were diluted to 4 ng/μl and pooled for sequencing on a HiSeq X platform (Illumina, San Diego, CA, USA) with 150-bp paired-end reads.

Low-quality reads and adaptor sequences were first removed with fastQC and fastp to generate clean reads (19). Subsequently, clean reads were mapped to the reference genome GRCh38 (Ensembl version 92 gene annotation) with STAR (20). After that, expression matrix files were generated based on gene counts and unique molecular identifier (UMI) counts that were acquired by featureCounts software (21).

### Quality Control, Dimension Reduction, and Clustering of the Single-Cell RNA Sequencing Data

Raw reads from each patient were processed to generate gene expression profiles using a celescope1.3.0 pipeline. Briefly, after filtering read 1 without poly T tails, valid cell barcode and UMI were extracted. Adapters and poly A tails were trimmed (fastp V1) before aligning read 2 to GRCh38 with ensemble version 92 gene annotation (fastp 2.5.3a and featureCounts 1.6.2) (21). Reads with the same cell barcode, UMI, and gene were grouped together to calculate the number of UMIs per gene per cell. The UMI count tables of each cellular barcode were used for further analyses. Before further analyses, cells were filtered by UMI counts, gene counts, and the mitochondrial content ratio. Only cells with UMI counts below 30,000, gene counts between 200 and 5,000, and the mitochondrial content below or equal to 50% were retained. After filtering dimension reduction and clustering were applied using Seurat v3.1.2 (22). Specifically, NormalizeData and ScaleData functions were utilized for the normalization and scaling of all gene expressions. Then, the expression matrix of each patient was integrated, principal component analysis (PCA) was performed with the top 2,000 variable genes that were selected by FindVariableFeautres(), and the top 20 principal components were used to separate cells into multiple clusters with FindClusters(). After that, the Uniform Manifold Approximation and Projection (UMAP) algorithm was applied to visualize cells in a two-dimensional space.

### Differentially Expressed Gene Analysis

To identify differentially expressed genes (DEGs) of each cluster, Seurat v3.1.2 FindMarkers() was utilized to select genes based on Wilcoxon likelihood-ratio test with default parameters. DEGs were defined as genes that are expressed in more than 10% of the cells in a cluster and with an average log_2_(Fold Change) of greater than 0.25.

### Cell Type Annotation

The cell type of each cluster was annotated based on the expression of canonical markers found in the SynEcoSys database (Singleron Biotechnology). Seurat v3.1.2 DoHeatmap(), DotPlot(), and Vlnplot() were used to generate heatmaps, dot plots, and violin plots to display the expression of the markers used to identify different cell types, respectively.

### Single-Cell RNA Sequencing-Based Copy Number Alteration Detection

The copy number alterations (CNAs) in cancer cells were detected with InferCNV package (23), with non-malignant immune cells as baselines. Genes expressed in more than 3 cells were sorted based on their loci on each chromosome. The ceiling of the relative expression values was set as 1.5 SDs from the residual-normalized expression values, and the relative expression values were centered at 1. The relative expression on each chromosome was smoothened using a slide window size of 101 genes to remove the effect of gene-specific expression.

### Pathway Enrichment Analysis

To investigate the potential functions of DEGs, the “clusterProfiler” R package version (24) was used to perform Gene Ontology (GO), Kyoto Encyclopedia of Genes and Genomes (KEGG), and Reactome analysis. Adjusted p (p_adj) values of less than 0.05 were used to define significantly enriched pathways. Referred GO gene sets included molecular function (MF), biological process (BP), and cellular component (CC) categories. Gene set enrichment analysis (GSEA) was also performed in the macrophage subclusters Mac3 and Mac4.

### Coupled Non-Negative Matrix Factorization Analysis

With the use of the cNMF algorithm, the genes of targeted cell types were first filtered, and the number of meta-programs was confirmed according to the statistical stability and the prediction error rate. Then, the meta-programs were extracted, and the score of each program was calculated for each cell.

Also, the Jaccard similarity coefficient was used to compare the transcriptional similarity between the meta-programs and particular cell types. The Jaccard similarity coefficient was calculated using the top 100 marker genes of each cell type and the top 100 genes of each meta-program.

### Trajectory Analysis

To map the differentiation/conversion of particular cell types, pseudotime trajectory analysis was performed with Monocle2 (25). To construct the trajectory, Seurat v3.1.2 FindVairableFeatures() was used to select highly variable genes from clusters, and dimension reduction was performed with DDRTree(). Finally, the trajectory was visualized by plot_cell_trajectory().

### RNA Velocity

For the RNA velocity analysis, the BAM files containing cancer cells from each patient were analyzed with the velocyto (26) and the scVelo (27) in python with default parameters. The reference genome used was GRCh38 (Ensembl version 92 gene annotation). To ensure visualization consistency, “runUMAP” function of Seurat was applied to plot the corresponding cell populations.

### Single-Cell Entropy Analysis

To evaluate the stemness of cells, the entropy of gene expression was calculated based on single-cell expression profiles with SLICE (version 0.99.0) (28). ERCC spike-ins and ribosomal genes were removed, and SLICE object was created to perform the bootstrap calculation of single-cell gene entropy values using the getEntropy() function.

### Violin Plots of Differentially Expressed Genes in Focus Subclusters of Cancer Cells

Violin plots were utilized to demonstrate the enhanced expression of genes in specific subpopulations after the re-clustering of cancer cells. The focus subcluster was defined as the dominant subcluster with the highest proportion in the cancer cells of each patient, which included the C3 subcluster for the P01 cancer cells, the C6 subcluster for the P02 cancer cells, and the C4 subcluster for the P03 cancer cells. The gene expressions in the focus subcluster and other subclusters were visualized in violin plots using VlnPlot() function.

### Cell–Cell Interaction Analysis

To analyze the cell–cell interaction, the CellPhoneDB (29) was utilized based on known receptor–ligand pairs. To calculate the null distribution of average ligand–receptor expression levels in the interacting clusters, the cluster labels of all cells were randomly permuted 1,000 times. The threshold of cells expressing within each cluster to 0.1 was then set, and the significant interaction pairs whose p-value <0.05 was visualized with the plot function embedded in the CellphoneDB.

### Immunofluorescence

All tissues were fixed in 4% formaldehyde, then embedded in paraffin, and cut into sections of 5-µm thickness for staining with H&E and immunostaining. After being deparaffinized and rehydrated and antigen was retrieved and blocked by standard techniques, sections were then incubated overnight at 4°C with primary antibodies at the following dilutions: CD68 (1:100, Servicebio, Gent, Belgium), NAPSA (1:100, ABclonal, Woburn, MA, USA), CD206 (1:3,000, Servicebio), G3BP2 (1:100, Affinity Biosciences, Cincinnati, OH, USA), SIRPA (1:50, SAB, Nanjing, China), HLA-F (1:8000, ProteinTech, Chicago, IL, USA), CD47 (1:100, HUABIO, Woburn, MA, USA), CD44 (1:100, Bioss, Woburn, MA, USA), and SPP1 (1:100, HUABIO).

## Results

### Distinct Pathological Features of Ground-Glass and Solid Components in Same Mixed Ground-Glass Opacities

We enrolled five non-smoking mGGO patients with clear GG/S margin. By synchronizing the identification of CT and tissue section appearance, different components of mGGO were able to be distinguished among three of them, followed by scRNA-seq (**Supplementary Figure 1A** and **Supplementary Table 1**).

The images of the mGGOs and their pathological sections revealed different features in the GG and S component areas. The GG components are focal nodular areas of alveolar epithelial cells in a lepidic growth pattern, thickened alveolar walls with an enlarged air-containing cavity, and infiltrated immune cells (**Supplementary Figure 1B**). The S components could be relatively consistently characterized by the absence of organized fibrous structures and obvious alveolar tissues and presented large areas of disorganized fibrous necrosis, third lymph nodes, tumor cell infiltration, and malignant gland tissue (**Supplementary Figure 1C**). Tissues were dissected and then digested into single-cell solution, and then single cells were counted. Tissue density was continuous in GG regions, whereas S areas were distinct (**Supplementary Figure 1D**).

### A Single-Cell Landscape of Paired Ground-Glass and Solid Components From Mixed Ground-Glass Opacities

**Figure 1:**
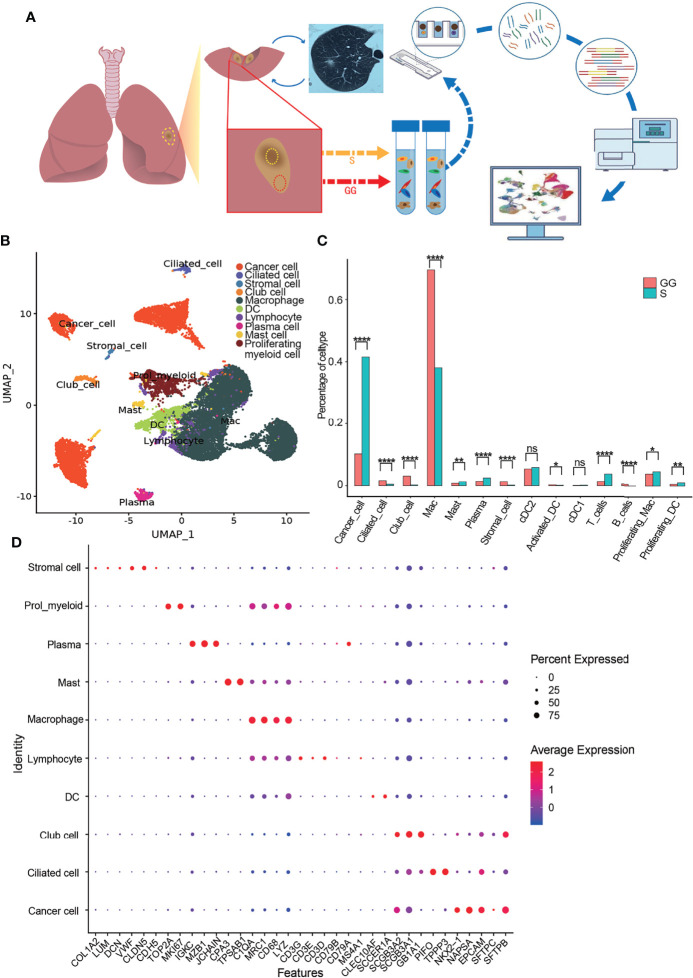
Sampling, sequencing, and clustering from mGGO. **(A)** Sampling and sequencing workflow. **(B)** Cell atlas of mGGO shown by UMAP; each dot corresponds to a single cell, and clusters are labeled with names and colored. **(C)** Differences of cluster proportion between S component and GG component of mGGO; chi-squared test was used for analysis. **(D)** Bubble plot shows mean expression level of marker genes (x-axis) for each cell type (y-axis); dot size, proportion of cells expressed the gene; dot color, average expression level among cells. mGGO, mixed ground-glass opacity; UMAP, Uniform Manifold Approximation and Projection; S, solid; GG, ground glass. * P<0.05, ** P<0.01, **** P<0.0001, ns non-significance.

To characterize the cellular dynamics in mGGO components, a total of 19,391 isolated single cells (8,635 from the GG components and 10,392 from the S components) were obtained from three patients diagnosed with Invasive Adenocarcinoma (IAC) and were subjected to UMAP clustering analysis (**Figure 1A**). All cells were classified into nine major cell types (**Figure 1B**). Based on the expression of cell-type-annotation markers from the SynEcoSys database, we found that the cells are comprised mainly of clusters of cancer cells (GG components, 975; S components, 4,629), macrophages (GG components, 5,888; S components, 3,705), dendritic cells (DCs) (GG components, 487; S components, 602), T cells (GG components, 115; S components, 363), B cells (GG components, 50; S components, 3), plasma cells (GG components, 116; S components, 240), mast cells (GG components, 68; S components, 124), club cells (GG components, 257; S components, 27), and ciliated cells (GG components, 134; S components, 51) (**Figures 1C, D**). Moreover, we identified a cluster annotated as the proliferating myeloid cells, which were composed of macrophages (GG components, 327; S components, 450) and DCs (GG components, 51; S components, 102) in proliferation, and the proportions of the S components were slightly higher than those of GG areas (**Figure 1C** and **Supplementary Figure 2A**).

The most abundant cells of mGGOs were observed to be macrophages and cancer cells, occupying nearly 50% of the cells in the GG and S component samples, respectively (**Figure 1C** and **Supplementary Figure 2B**), and this was further confirmed by immunofluorescence staining (**Supplementary Figure 2C**). Moreover, we confirmed that DCs were the third major cell type, dominated by conventional DCs (cDCs). No significant differences were observed in the numbers of cDC subtypes between the S and GG components. The proportion of lymphocytes was about 5% in all samples, dominated by CD8+ T cells and plasma cells, indicating activated adaptive immune responses (**Supplementary Figure 2D**). In addition, we found discrepancies in cell composition between the GG and S component samples. In the S components, the epithelial cell types were largely composed of cancer cells, while a certain amount of normal club cells and ciliated cells were retained in the GG components, which were consistent with the previous report (15). These cellular compositions demonstrated both the common and distinct characteristics between the GG and S components.

Cancer cells and macrophages were the dominating cell types constituting the mGGOs, and we hypothesized that the activities of these two cell types were key to unraveling the mechanisms underlying the development of mGGOs.

### Cancer Cells From the Solid Components and Ground-Glass Components Exhibit Different Transcriptional Features

**Figure 2:**
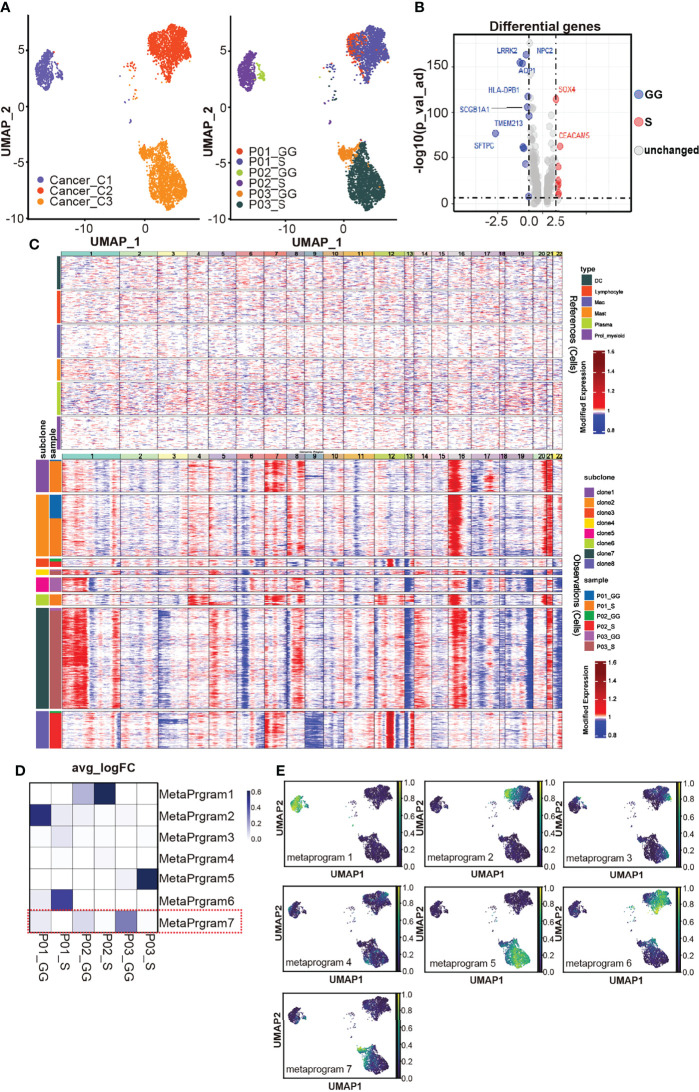
Distinct transcriptome signatures of cancer cells in different components. **(A)** The UMAP visualization of cancer cells: the left graph shows that they are clustered into three subclusters, and the right graph labels these cells by sample sources. **(B)** The volcano plot reveals differentially expressed genes in S component and GG component. Threshold has been set as 10^−5^ for p_val_adj and 0.75 for avg_logFC. **(C)** Hierarchical clustering of the chromosomal gene expression pattern separating cancer cells from non-malignant immune cell clusters. Each row represents a sample, and each column represents a single cell. **(D)** The UMAP visualization shows the expression level of gene sets within each meta-program clustered by the cNMF in cancer cell clusters. **(E)** The heatmap shows the expression level of gene sets within each meta-program (row) by sample (column). The red box marks GG component-specific meta-program. UMAP, Uniform Manifold Approximation and Projection; S, solid; GG, ground glass; cNMF, coupled non-negative matrix factorization.

Cancer cells were identified with canonical markers of LUAD and alveolar epithelial cells (e.g., *SFTPB*, *SFTPC*, *EPCAM*, *NAPSA*, and *NKX2-1*). A total of 4,905 cancer cells (4,040 originated from the S component samples and 865 initiated from the GG component samples) were clustered into three distinct subsets, each of which consisted almost exclusively of cancer cells with single-patient origin. Within each subgroup, there was a more pronounced difference between cancer cells of the S component versus GG origin (**Figure 2A**). This suggests a predominance of tumor heterogeneity between patients, while cancer cells from different radiological components exhibit milder intratumoral heterogeneity. Cancer cells in the S components upregulated *SOX4* and *CEACAM5* and downregulated pro-inflammatory factors such as *SCGB1A1* and *SFTPC* in comparison with cancer cells in the GG components (**Figure 2B**). The Reactome pathway enrichment analysis showed that the interleukin-related pathway, receptor tyrosine kinase (RTK) signaling, VEGFR2-mediated proliferation regulatory signals, and interferon alpha/beta signaling pathway were upregulated in cancer cells located in the S components. Moreover, the MAPK pathway, TNF signaling pathway, and apoptosis-related pathway were also enriched by KEGG pathway analysis (**Supplementary Figure 3A**). In comparison with cancer cells in the GG components, downregulated pathways include interferon gamma signaling pathway, surfactant metabolism, and lysosome-related pathway (**Supplementary Figure 3B**). The copy number variations (CNVs) were higher in cancer cells relative to immune cells (**Figure 2C**). Cancer cells were classified into 8 sub-clones based on the CNVs. Each patient harbored unique sub-clones, while some sub-clones (clones 1, 2, 3, and 8) were shared by cancer cells in different components of one individual. To obtain transcriptional signatures of cancer cells from different samples, we performed the cNMF consensus clustering of RNA-seq data and identified seven meta-programs of cancer cells. The meta-program scores were heterogeneous in S component samples, while similar patterns among the GG components were observed, especially the meta-program 7 (**Figures 2D, E**). The meta-program 7 gene signature included genes encoding surfactant-associated proteins A1, A2, B, C, and D (*SFTPA1*, *SFTPA2*, *SFTPB*, *SFTPC*, and *SFTPD*), cell adhesion factors, lysosomal homeostasis-related genes, and factors regulating cell growth cycle or apoptosis. We found that meta-program 7 included genes of extracellular matrix receptor proteins, such as integrin superfamily members including *ITGB6* and *ITGA2*, as well as *CD44* and *TNC* (**Supplementary Table 2**), indicating that it might influence tumor progression by mediating signaling of immune modulators and regulating bindings and adhesion to collagen fibers (30, 31). In a nutshell, these data imply that the cancer cells are more heterogeneous with high expression levels of cancer biomarkers in the S component samples, and it might be flawed to find a uniform molecular signature for these cancer cells. The cancer cells in the S component samples were more heterogeneous and showed more obvious expressions of cancer biomarkers. The cancer cells in the GG component samples, however, highly expressed surfactant-associated proteins and exhibited stress-related features.

### Cancer Cells in the Solid and Ground-Glass Components Differ in Heterogeneity and Stemness, But Both May Contribute to the Tumor Development

**Figure 3:**
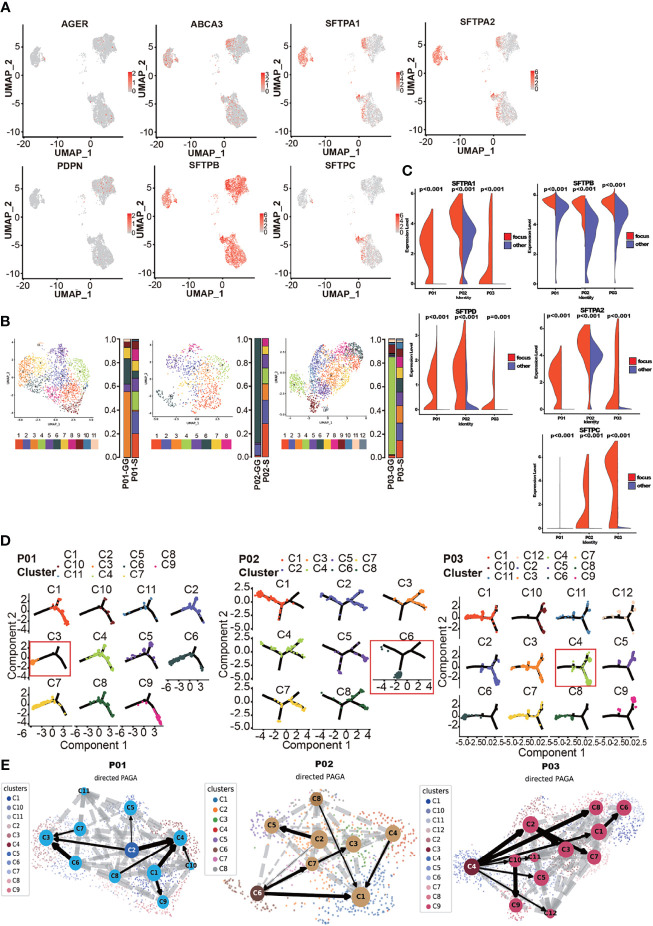
Origin of cancer cells in solid part of mGGO. **(A)** The gene plot of published AT1 (AGER and PDPN) and AT2 (ABCA3, SFTPA1, SFTPA2, SFTPB, and SFTPC) markers expressed in cancer cells; red dots are cells that expressed these genes. **(B)** Subclustering of cancer cells by each patient; each color represents a subcluster of cancer cells, and the proportion of each subcluster is displayed on the bar graph on the right. **(C)** The violin plot reveals differential expression of surfactant family between ground-glass (GG) and solid (S) components growth privilege cancer subcluster (labeled as focus) and other clusters (labeled as other). Wilcoxon rank-sum test was used for analysis. **(D)** The UMAP visualization of cancer subclusters trajectory used monocle by sample. Each subcluster is plotted individually, and the red box marks the GG/S component growth privilege cancer subcluster. **(E)** The PAGA embedded with RNA velocity reveals the possible trajectory of cancer subclusters. Subclusters highlighted with darker node backgrounds are the assuming origin cluster based on RNA velocity. mGGO, mixed ground-glass opacity; UMAP, Uniform Manifold Approximation and Projection.

To investigate the grade of cancer cells, we explored alveolar type I (AT1) and alveolar type II (AT2) cells with annotated markers, including *PDPN*, *AGER*, *ABCA3*, and *SFTP* gene families (32, 33) (**Figure 3A**). The AT1 markers *PDPN* and *AGER* were sporadically expressed, indicating a low ratio of AT1-like cells. AT2 markers *SFTPB* and *ABCA3* were simultaneously expressed in a large proportion of cancer cells. Most cancer cells in the GG components expressed *SFTPA1* and *SFTPA2*, although cancer cells in the S components of different individuals had diverse distribution patterns. SFTPC is expressed in a higher proportion of cancer cells in the GG component states. To sum up, AT1-like cells were rarely found in mGGO samples. AT2-like cells varied among patients. Interestingly, we found that the majority of cancer cells from P01-GG expressed all AT2 markers, which strongly indicated their origin from AT2 cells.

Taking the consideration of CNVs where cancer cells between different components of mGGO shared similar structural genomic rearrangements within each patient, the homogenous genesis of the GG and S components was suggested. To explore whether there was a developmental relationship between the S and GG components, we clustered the cancer cells from individuals (**Figure 3B**). We discovered that cancer cells seemed less heterogeneous and that each patient possessed a dominant cancer cell subcluster (P01-C3, P02-C6, and P03-C4) in the GG component. In contrast, in the S component samples, cancer cells demonstrated a mixed composition of multiple subclusters without any dominant subpopulation, which was consistent with the pathological features of LUAD (34).

We then analyzed the DEGs of the dominant subclusters in the GG component samples. Similar to the overall transcriptional profile of cancer cells in the GG component samples, surfactant-associated proteins (*SFTPA1*, *SFTPA2*, *SFTPB*, *SFTPC*, and *SFTPD*) were highly expressed, and the expression level was higher than that of other cancer cell subclusters of the GG component samples (**Figure 3C**).

To investigate the developmental relationship between cancer cells from different pathological areas of mGGOs, we conducted the trajectory analysis of cancer cells from each of the three patients. The dominant subclusters of cancer cells in the GG components were generally located at one end of the trajectory, while the distributions of cancer cells were dispersed in the S components (**Figure 3D**). This indicates that most of the cancer cells in the GG components were in a similar state of the putative evolutionary process, whereas cancer cells in the S component were scattered along the trajectory and showed branching evolutionary architectures. Furthermore, we also mapped the developmental trajectories with the RNA velocity analysis for cancer cells in each patient, so as to infer the cancer cell subcluster in the initial state (27). The results showed that the initial state cancer cell subclusters in two of the three patients (P02 and P03) were the dominant subclusters of the GG components, while the dominant cancer cell subcluster (P01-C2) in the GG component of patient P01 was the second to the initial state (**Figure 3E**). Moreover, tumor-promoting genes such as *HMGB3* and *IGFBP2* were highly expressed in P01-C2, which implied a poorly differentiated subpopulation of malignant cancer cells (35). In addition, the stemness of cancer cells in the S component samples was slightly higher than that in the GG components (**Figure S3C**). Taken together, our results suggest that the cancer cells could originate from either GG foci cancer cells or solid foci cancer cells.

### Diversity of Macrophages Within Different Components in Mixed Ground-Glass Opacity

**Figure 4:**
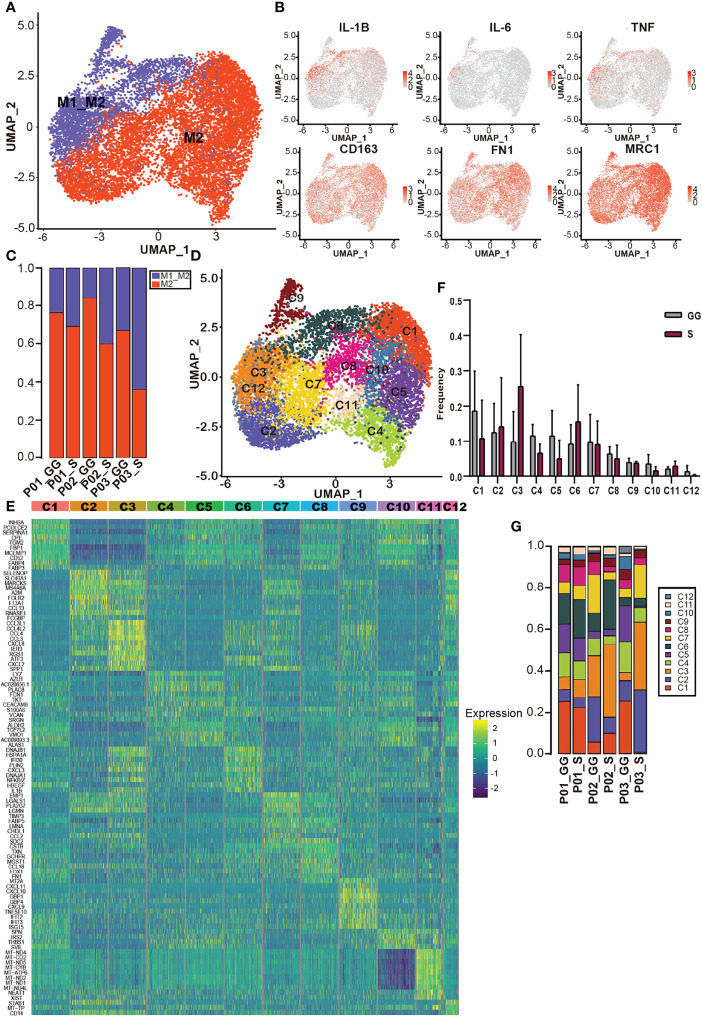
Diversity of macrophages within different components in mGGO. **(A)** The UMAP of M1_M2 macrophages (blue) and M2 macrophages (red) distribution. **(B)** The UMAP shows expression patterns of M1 macrophage markers IL-1B, IL-6, and TNF and M2 macrophage markers CD163, FN1, and MRC1. **(C)** Proportions of M1_M2 macrophage (blue) and M2 macrophage (red) in different samples. **(D)** The UMAP visualized 12 macrophage subclusters. **(E)** The heatmap reveals distinct transcription patterns among macrophage subclusters; each row represents a gene, and each column represents a macrophage. **(F)** Proportions of 12 macrophage subclusters (x-axis) reveal distinct distributions between GG component (gray) and S component (red) in mGGO. **(G)** Proportions of macrophage subclusters in different samples. mGGO, mixed ground-glass opacity; UMAP, Uniform Manifold Approximation and Projection; GG, ground glass; S, solid.

According to the expression of annotated marker genes *LYZ*, *CD68*, *MRC1*, and *MARCO*, we identified a total of 9,593 macrophages (**Figure 1D**). Further, we identified two macrophage types with canonical marker gene expression (*IL-1B*, *IL-6*, and *TNF* for M1 macrophages, and *CD163*, *MRC1*, and *FN1* for M2 macrophages), and we confirmed that M2 macrophages were predominant in the mGGOs, and a small portion of cells expressed both M1 and M2 markers (referred as the M1–M2 type) (**Figures 4A, B**). We noticed that the proportion of M1_M2 macrophage was higher in the S components (**Figure 4C**).

Graph-based clustering gave rise to 12 macrophage subclusters (**Figures 4D, E**). Mac1–Mac6 were the most abundant subclusters within the samples (**Figure 4F**). Among these clusters, Mac1-*INHBA* ^hi^, which upregulated *FABP* and *CD52*, and Mac2-*FOLR2* ^hi^, with upregulation of *CCL13*, showed diverse distribution in the S/GG components among different individuals (**Figure 4F**). Mac3-*SPP1* ^hi^ and Mac4-*LYZ* ^hi^-*SPN* ^lo^ were enriched in the S and GG components, respectively. Mac6-*IL-1B* ^hi^ and Mac5 also showed similar but weaker distribution bias in different components (**Figure 4G**). Interestingly, the transcription features of Mac4 and Mac5 were similar. For example, they both upregulated *LYZ* and *AZU1*, suggesting their common roles in the antimicrobial process. However, Mac3-SPP1^hi^ was characterized by a high expression of *CCL3*, *CCL4*, *CXCL2*, and *CXCL8*, and so did Mac6-*IL-1B* ^hi^, with upregulation of *CXCL3*, indicating an active function of chemoattraction and recruitment of inflammatory cells (36). Macrophages with high expression of SPP1 were also found to be one of the common phenotypes in lung cancer (37). Taken together, these results depict the distinct distribution of macrophage subclusters within different components of mGGO.

**Figure 5:**
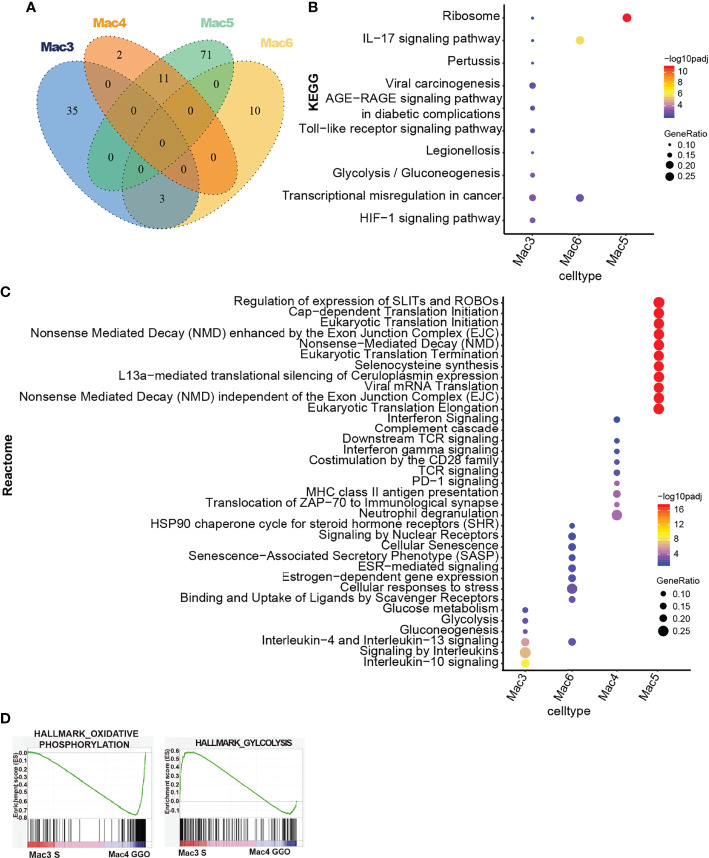
Pathway enrichment of S/GG component-dominant macrophage subclusters. **(A)** The Venn diagram shows overlapping significantly upregulated pathways in Mac3, Mac6, Mac4, and Mac5, **(B, C)** The bubble plot shows significantly enriched KEGG and Reactome pathways in Mac3, Mac6, Mac4, and Mac5. The colors of bubbles represent the values of significance, and the sizes represent the number of genes enriched in the pathway. **(D)** The GSEA using pre-ranked differentially expressed genes between Mac3 _S component versus Mac4 _GG component were compared with hallmark gene sets. The pre-ranked gene set was defined by function of the FindMarkers in Seurat. The enrichment of the oxidative phosphorylation pathway and glycolysis pathway is shown on the graph. S, solid; GG, ground glass; KEGG, Kyoto Encyclopedia of Genes and Genomes; GSEA, gene set enrichment analysis.

We conducted the KEGG, GO, and Reactome pathway analyses on macrophage subclusters. No shared pathways were found between the Mac3 and Mac6 group and the Mac4 and Mac5 group, while both groups upregulated a few pathways in common within-group (**Figure 5A**). IL-4, IL-10, and IL-13 signaling, along with significantly elevated tumor-associated transcription factor abnormalities, upregulation of IL-17 and Hif-1a pathways, and glucose metabolism pathway, was enriched in Mac3 (**Figures 5B, C** and **Figure S4A**), which pointed to M2 characteristics. Furthermore, AGE-RAGE and Toll-like receptor (TLR) signaling pathways were also upregulated, accompanied by the upregulation of viral oncogenic pathways (**Figure 5B**), which pointed to M1 characteristics. Such transitional transcriptional features with both M1 and M2 characteristics could also be found in another subcluster, Mac6, which was also enriched in the S component samples.

Furthermore, although glucose metabolism was upregulated in Mac3, we found that the expression level of genes that related to oxidative phosphorylation was higher in Mac4 cells than Mac3 cells, which were mainly derived from the GG and S component samples, respectively (**Figure 5D**). The metabolic requirement of cell proliferation is different in normal cells compared to cancer cells (38), and recent studies have found that in the tumor microenvironment (TME), myeloid cells have the highest glucose uptake rate (39). Therefore, these data indicate a possible shift in the glucose uptake and utilization during the early-stage tumorigenesis of lung cancer.

Taken together, our data depicted various ecosystems of macrophages in the mGGO lesions and distinct features of macrophages enriched in different components of mGGO.

### The Macrophages in the Solid Component Samples Upregulated NF-κB-Related Signaling

**Figure 6:**
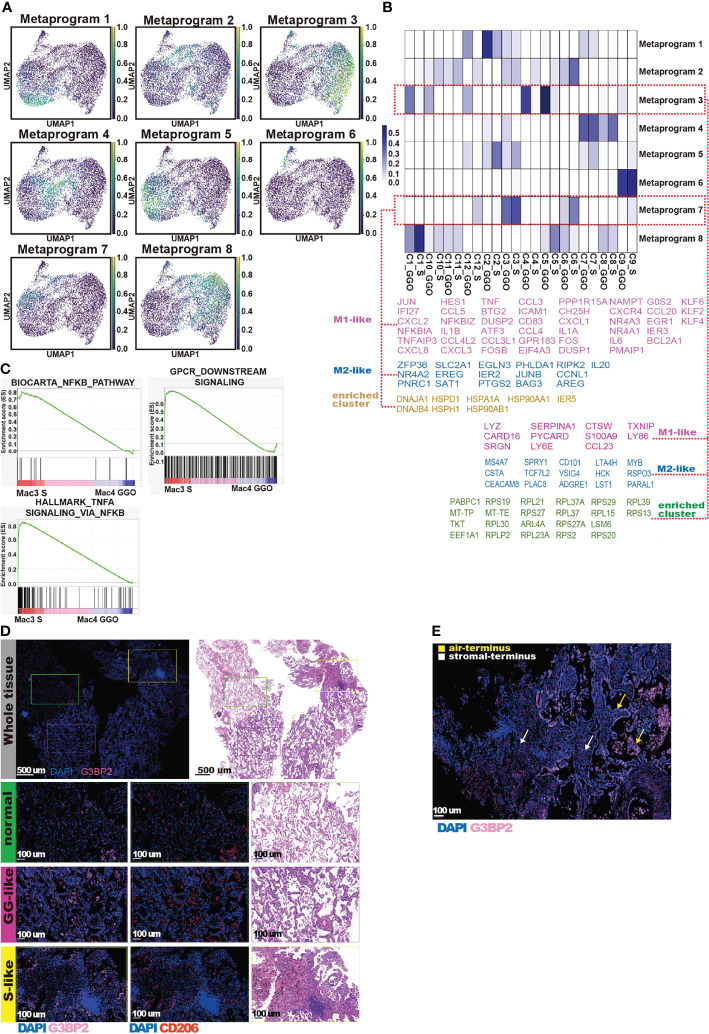
cNMF and IFA reveal different signatures of macrophages between GG component and S component. **(A)** The UMAP shows meta-program of macrophage genes clustered by the cNMF; the shades of the colors represent proportions of cells expressing the meta-program. **(B)** The heatmap reveals the expression level of meta-program (row) in each sample (row); GG dominant meta-program (up) and S dominant meta-program (down) are marked with red boxes. We also list M1-like genes, M2-like genes, and enriched gene sets with similar functions in each highlighted meta-program. **(C)** The GSEA using pre-ranked differentially expressed genes between Mac3 _S component and Mac4 _GG component were compared with hallmark gene sets, Biocarta gene sets, and GPCR gene sets. The pre-ranked gene set was defined by function of the FindMarkers in Seurat. We show the enrichment of NF-κB pathway (top left), NF-κB-initiated TNF-alpha signaling pathway (bottom left), and GPCR pathway (right). **(D)** G3BP2 expression in nlung, GG-like, and S-like tissue. The “Whole Tissue” panel shows the overall region of an “mGGO looking” area with IFA and H&E stains. A typical field of nlung, GG-like, and S-like was selected using green, pink, and yellow boxes, respectively. Scale bars, 500 μm. The zoomed-in version of each field is below the panels (row). Each panel, from left to right, shows the image of DAPI (blue) + G3BP2 (pink), DAPI (blue) + CD206 (red), and H&E stains. Scale bars, 100 μm. **(E)** IFA shows the high expression level of G3BP2 (red) in lepidic growth cancer cells and macrophages in the alveolar cavity (yellow arrow) compared with those infiltrated into stromal cells (white arrow). cNMF, coupled non-negative matrix factorization; IFA, immunofluorescence assay; GG, ground glass; S, solid; UMAP, Uniform Manifold Approximation and Projection; GSEA, gene set enrichment analysis.

Next, we performed the cNMF clustering analysis and identified 8 meta-programs of macrophages (**Figures 6A, B**). Meta-program 7 had relatively high scores in Mac3 and Mac6. Interestingly, the meta-program 7 scores were higher in all M1–M2 state subclusters (Mac3, Mac6, Mac9, and Mac12). The meta-program 7 gene signature mainly consisted of heat shock protein family genes, GPCR signaling pathway-related genes, inflammatory cytokine genes, and tumor suppressor genes (**Figure 6B** and **Supplementary Table 3**). We noticed that *NFκB* repressing factors such as *NFκBIA* and *NFκBIZ* were also included in this meta-program. We compared the enrichment of the NFκB pathway and the pathways downstream of NFκB in the Mac3 cells from the S component samples and the Mac4 cells from the GG components using the GSEA and found that these pathways were all enriched in the S component Mac3 macrophages (**Figure 6C**). Therefore, we speculate that the *NFκB* repressing factors in the S component Mac3 macrophages are negative-feedback regulations caused by the overactivation of *NFκB*. In addition, genes related to *AP-1* and *Bcl-2*, such as *FOS*, *JUN*, and *BCL2A1*, were also enriched in the meta-program 7 (**Figure 6B**). These genes are essential regulators of the direction of macrophage polarization (40, 41). Taken together, these features demonstrate the association of the atypical polarization feature of macrophages with the upregulation of meta-program 7, especially in the S components of mGGOs.

### The Macrophages in the Ground-Glass Component Samples Show a Gain of Function for Dealing With Infection and Cell Mutation

Similarly, we also investigated the macrophage subclusters of the GG component samples. In subcluster Mac4, the pathway enrichment analyses showed that the pathways related to complement cascade, ZAP-70 signaling pathway, and MHC class II antigen presentation were upregulated (**Supplementary Figure 5C**). This suggests that Mac4 is probably involved in clearing pathogens/antigens and presenting them to the adaptive immune system. Meanwhile, in Mac1 and Mac5, which constituted a large proportion of macrophages in the GG components, with similar features, ROBO/Slit signaling pathway, nonsense-mediated mRNA degradation pathway (NMD), and aberrant translation pathway associated with viral infection (**Figure 5C** and **Supplementary Figure 4A**). Upregulation of the NMD pathway was associated with the sensation of cellular stress (42) and enhancement of antitumor immune surveillance (43). In addition, both the NMD and ROBO/slit pathways are associated with viral infections (44), indicating that the development of GGOs is probably associated with infections. Interestingly, the cNMF analysis of macrophages showed that the subclusters highly represented in the GG components (C1, C4, C5, C10, and C12; **Figure 4F**) were all highly associated with the meta-program 3 (**Figures 6A, B**). This gene set contained a large number of ribosomal protein genes as well as genes involved in RNA processing and maturation, suggesting active translational activities in macrophages in the GG components (**Figure 6B**). Due to the enrichment of the NMD and the active RNA processing and translation-related functions in pathway analysis and cNMF clustering, we speculate that macrophages from the GG components may orchestrate pathways to disrupt protein translation. We examined the expression of G3BP2, which encodes core components of stress granules (SGs). We found that the G3BP2 level was significantly elevated in the GG components of mGGOs compared with that of normal lung tissue, and the expression level was higher in the air-end tissues than in the stromal end tissues of mGGOs (**Figures 6D, E**). The formation of SGs has been discovered during tumorigenesis and can be induced by viral infections as well as the transcriptional/translational burden caused by mutations (45, 46). In a nutshell, macrophages in the GG components were under the highest level of stress in comparison with normal lung tissues and the S components.

### Cancer Cell-Related Interactions Suggest Different Tumor Development Patterns in Different Mixed Ground-Glass Opacity Components

**Figure 7:**
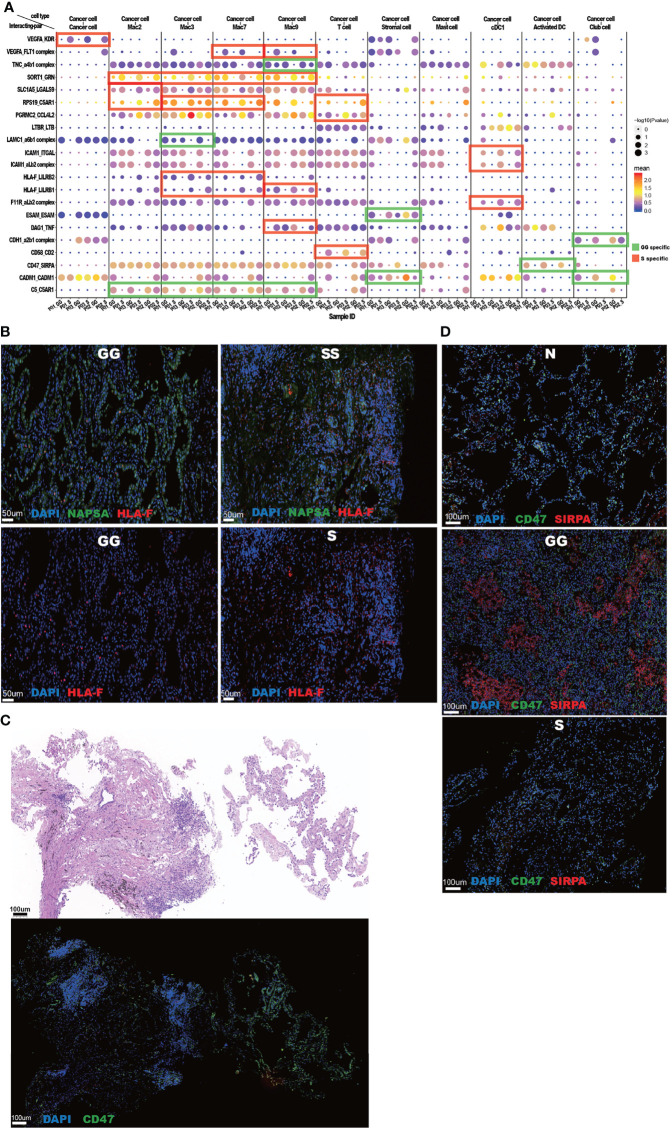
Cancer cell-related interactions suggest different tumor development patterns in different mGGO components. **(A)** The bubble plot of cancer cell-related ligand–receptor pairs reveals S and GG preferences. The x-axis shows cell subsets, and the y-axis shows ligand–receptor pairs. Bubbles are annotated by −log_10_(P) (size) and mean expression level of ligand–receptor pairs (color). S and GG preference ligand–receptor pairs are highlighted by red and green shades, respectively. **(B)** IFA shows upregulation of HLA-F in cancer cells from S components in comparison with those from the GG components. Scale bars, 50 μm. **(C)** Consequent slices of mGGO show the S components (left) and the GG components (right) from an mGGO tissue. IFA on the bottom shows upregulation of CD47 in cancer cells from the GG components. Scale bars, 100 μm. **(D)** IFA shows the dramatic expression level of CD47 (green) and SIRPA (red) in the GG components (middle, labeled as GG) compared to normal lung (top, labeled as N) and S components (bottom, labeled as S). Scale bars, 100 μm. mGGO, mixed ground-glass opacity; S, solid; GG, ground glass; IFA, immunofluorescence assay.

Due to the obvious difference in gene expression patterns among cancer cells in different mGGO components, we hypothesize that such discrepancies could lead to distinct interactions between cancer cells and the TME. We investigated cancer cell-specific interaction pairs in different mGGO components by CellphoneDB (**Figure 7A**). The S component samples were enriched with angiogenesis-related interactions, with high expression of *VEGFA* and *FLT1* (encoding VEGFR1) in cancer cells and macrophages, respectively. An *in vitro* study has verified that M2-type macrophages highly expressing *FLT1* assist epithelial cells in rapid angiogenesis (47). Furthermore, *HLA-F* was upregulated in cancer cells from the S components, which was shown to be correlated with the upregulation of *LILRB1/LILRB2* in macrophages (**Figure 7B**). In addition, compared to cancer cells in the GG components, cancer cells in the S component samples exhibited more significant potential interactions with DCs, mainly *via* adhesion molecules such as *ICAM1* and *F11R*. At the same time, the adhesion molecule of CD58 that interacted with T-cell receptor CD2 was highly expressed in cancer cells, indicating higher immunogenicity in the S component samples, which activated T cells for its cytotoxicity (48). The highly expressed *TNF* in macrophages in the S components was associated with upregulated *DAG1* in cancer cells, which regulated cell growth and apoptosis (49). Taken together, the interactions between cancer cells and immune cells showed an intricate regulation network in the S components, including immune-suppressive interactions such as *HLA-F_LILRB1*/*LILRB2 (* 50, 51) and *RPS19*_*C5AR1 (* 52) and immune-activating interactions such as *DAG1*_*TNF (* 53) and *CD58*_*CD2 (* 54). Also, immune cells especially macrophages are more likely to promote cancer development in S areas through highly specific interaction pairs such as *SORT1*_*GRN (* 55) and *PGRMC2*_*CCL4L2 (* 56).

Cancer cells in the GG components mostly interacted with stromal cells, club cells, and macrophages *via* adhesion molecules such as *ESAM*, *CADM1*, *CDH1*, and *LAMC*. In addition, *CD47* is specifically highly expressed in GG focal cancer cells, and the associated *SIRPA* is also highly expressed and correlated within many immune cells such as mature DCs, where CD47 acts as a classical immune escape molecule to block the maturation of immune cells and release immune-activating factors through the CD47–SIRPA axis (57). We further confirmed the alteration of *CD47* expression level between the GG and S components within a slide that contained both the GG and S components (**Figure 7C**). In addition, simultaneous upregulation of *CD47* and *SIRPA* was found to be a unique feature of the GG components in comparison with the S components and normal lung tissue (**Figure 7D**). It suggests a vital role of the CD47–SIRPA axis in the maintenance of the GG components. Taken together, our data demonstrated distinct interactions between cancer and immune cells of different components.

### Features of Cellular Interactions in Different Mixed Ground-Glass Opacity Components Reflect a Shift in the Immune Environment

**Figure 8:**
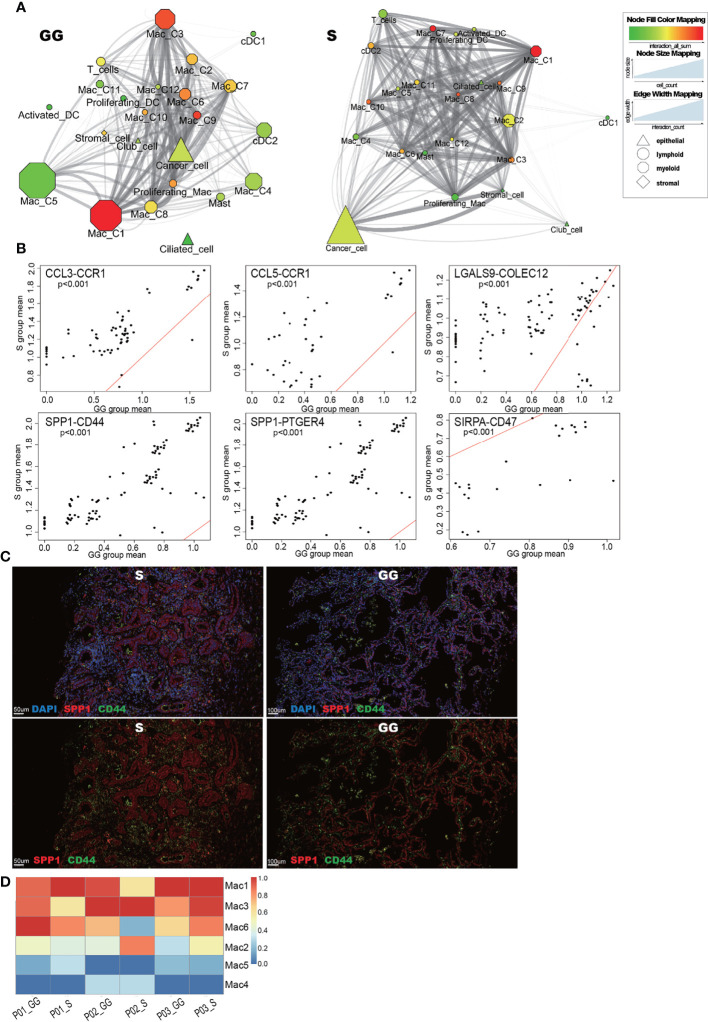
**(A)** The Cytoscape reveals different cell–cell interaction models between GG component (left) and S component (right). Organic layout based on a force-directed layout paradigm was used. Edges connect cell subsets (nodes) with significantly correlated ligand–receptor pairs (p < 0.05). Only high confidence of connection between a pair of cell subsets is visible (ligand–receptor pairs >60). Nodes were annotated by lineage (shape), cell numbers (size), and total interaction pairs (color). Edges’ width and transparency were annotated with the account of ligand–receptor pairs. **(B)** The scatter plot shows the GG- and S-specific macrophage-related interaction pairs. Each dot shows a pair of cell subsets interacting with the ligands and receptors, and each pair consists of at least one macrophage subset. The x-axis shows the mean expression value of ligand–receptor genes in the cell subsets in the GG component, and the y-axis shows the mean expression value of ligand–receptor genes in the cell subsets in the S component. Each cell pair satisfies the universal expression among samples. If dots are enriched above the red line (y = x), we defined the interaction pairs as S-specific. If they are enriched below it, we defined them as GG-specific interaction pairs. Wilcoxon rank-sum test was used for analysis. **(C)** IFA shows simultaneous upregulation of SPP1 (red) and CD44 (green) in S components (scale bar, 50 μm) in comparison with the GG components (scale bar, 100 μm). **(D)** The heatmap shows the total interaction of macrophage subsets (Mac1, 2, 3, 4, 5, and 6, y-axis) within each sample (x-axis).

To further demonstrate the rewiring of cell–cell interactions across different components, a force-directed graph was delineated by mapping the significant correlated receptor–ligand pairs onto cell subsets (**Figure 8A**).

The macrophage subclusters distributed differently not only in function but also in cellular interactions between the S component and GG components. The S component-enriching subclusters Mac3 and Mac6 showed stronger interactions compared with Mac4 and Mac5, which may be related to the more diverse functions of Mac3 and Mac6 (**Figure 7C**). In tissue sections, we found a large number of clustered macrophages in the enlarged alveolar space of the GG components, whereas in the S component samples, most macrophages were infiltrated within the extracellular matrix due to the absence of the alveolar space structure (**Figure 7D**). This difference in distribution may be one of the factors contributing to the differential behavior between the S and GG component macrophages.

The macrophage chemotactic protein SPP1 is highly expressed in infiltrating macrophages after tissue injuries, can sustain cancer cell survival, and promote angiogenesis (58, 59). Interestingly, Mac3 also expressed the highest level of SPP1, which is a markable macrophage subcluster within solid areas (60) (**Supplementary Figure 4C, D**). We found that highly expressed SPP1 was associated with upregulation of PTGER4 and CD44 in many cell types of the S components (**Figures 8B, C** and **Supplementary Table 4**). In addition, we observed the distinct expression levels of *CCL3*_*CCR1* and *CCL5*_*CCR1* interactions between the S and GG components, indicating the differences in the immunosuppressive microenvironment and inflammatory features between the S and GG components (61, 62) (**Figure 8B**). Furthermore, in the S component samples, TNF-related interactions such as *TNF*_*DAG1* were also upregulated in comparison with the GG components. We also noticed that the S component-enriched macrophage subclusters such as Mac3 and Mac6 displayed a significantly higher level of total interaction with other cell types in comparison with Mac4 and Mac5, which were both enriched in the GG components (**Figure 8D**). Overall, we believe that compared to the GG component samples, the S component samples exhibit more inflammation-related tumor characteristics, with macrophages playing a key role.

## Discussion

In this study, we depicted cell atlas and transcriptomic features of distinct areas in mGGO. The GG components are often concerned with non-invasive lesions, while the S components are usually the invasive parts. By comparing the single-cell profiles between the two areas, we explored the cellular and molecular changes in the very early stage of LUAD where the lesions become malignant. Inter-patient heterogeneity posed significant challenges in studying cancer genetics using human samples (15, 63). By evaluating samples from each participant independently, our study offset the huge individual differences. Only consistent differences shared by all participants were considered significant and discussed in detail.

Cancer cells have the largest heterogeneity when compared with other cell types. Cancer cells in solid regions display a more dispersed clustering pattern than those in GG regions, indicating that they were endowed with higher variability. This is in line with a prior study that found mixed GGO tumors showed higher heterogeneity than pure GGO tumors (15). This could be caused by the increased burden of mutations in solid areas (9). In GG regions, however, cancer cells usually have a dominant subcluster and several other minorities. Compared with other cancer subclusters, the dominant subcluster expressed higher SFTPs, particularly *SFTPA* and *SFTPD*. The maintenance of alveolar structures necessitates the presence of SFTP. Furthermore, some of the cancer cells in the GG components were still able to keep a strong AT2 identity, indicating that these cancer cells might directly originate from AT2 cells.

We also explored the evolution of cancer cells between different components by using monocle and PAGA trajectories embedded with RNA velocity. The dominant subclusters of the GG components are likely to be the origin of invasive cancer cells at an early stage of tumorigenesis in most cases. However, a subcluster in the initial state of putative evolution trajectory with high expression of *HMGB3* and *IGFBP2* was also found in the solid area. Both of the genes have been reported to correlate with cell proliferation and migration (64, 65). It suggests that the S components were more likely to give birth to stem cell-like malignant cells, which could differentiate into many other subtypes of cancer cells, leading to a great heterogeneity. Due to the individual differences among human samples and the intricate transformation process from normal tissue to early-stage malignancy, it is very hard to draw a clear trajectory by grouping samples. To address this problem, depicting the trajectory of each individual and seeking the common pattern is a feasible way. So far, our data show that the GG dominant subclusters were likely to be the origin of cancer cells in solid areas in two patients. However, we also found that some stem cell-like malignant cells in the S components might also be able to give birth to cancer cell subclusters similar to GG component states. If this is true, it would be interesting to demonstrate a mutual-evolvement model between the S and GG components, which might provide an explanation for the phenomena of the newly appearing GG components being found by the S components on occasion.

Cancer cells in the GG components have lower immunogenicity than those in solid areas given their high communication with immune cells through “don’t eat me” signals such as *CD47 (* 66). However, cancer cells in solid areas are characterized by increased expression of variant cancer-related genes such as *SOX4* and *CEACAM5 (* 67). The VEGFA signaling pathway, the MAPK pathway, and cancer transcriptional misregulation were all shown to be upregulated in cancer cells in solid regions, indicating a more invasive phenotype. Interestingly, these cells also appeared to be regulated by TNF signaling, with increased apoptosis and programming cell death, suggesting a conflicting mode of cancer growth and active immune surveillance in the S components.

By grouping macrophage subclusters based on their distribution in different areas, we defined 12 macrophage subclusters existing in mGGO. Mac3-*SPP1* ^hi^ was enriched in the S components with great consistency across different patients. Compared with other subclusters, Mac3 had significantly more differential expressed genes than those in GG areas. We hypothesized that it was specifically activated to exert some unique processes. These genes included transcription factor *ATF3* and its correlated downstream genes like CC and CXC chemokines such as *CCL3* and *IL-8 (* 68, 69), indicating its role in rewiring the immune microenvironment within the lesion. *SPP1* ^hi^ macrophages were also reported to proliferate in idiopathic pulmonary fibrosis (70). In addition, studies have indicated that *SPP1* ^hi^ macrophages could be a potential antitumor therapeutic target in several cancer types (69, 71). Our study suggests that *SPP1* ^hi^ macrophages could play an important role in contributing to the invasiveness of LUAD, pointing to a possible new target for preventing disease progression.

Cancer cells and macrophages in different locations seem to be differentially evoked by pressures. We clustered a subgroup with ribosome RNA upregulated in macrophages in the GG components and also showed translation correcting functions such as nonsense-mediated decay. Core proteins of stress granules G3BP2 were more converged in the GG components than those in normal lung and S components and tended to gather in air-exposed areas than the basal side. Stress granules are mRNPs stalled in translation initiation (72). Targeting these structures has been proven to be a feasible therapy for NSCLC based on a recent study (73). Our finding suggests that it might be effective to target pre-invasive lesions when it is still characterized by GGO.

Area-specific cell interactions were demonstrated in this study. We screened out significantly altered interactions shared by all enrolled patients. Cancer cells in solid areas discard tumor-suppressive adhesion interactions such as *CDH1*, *CADM1*, and *ESAM* with normal epithelial cells in comparison with those in the GG areas (74). Instead, they favor tumor-promoting adhesion interaction such as *ICAM1* to interact with immune cells such as DCs (75). Most of the S area-specific interactions imply tumor-promoting functions. Increased *CD44* and *PTGER4* in many cell types such as macrophages, DCs, cancer cells, and T cells were correlated to upregulated *SPP1* in macrophages; the activated interaction pairs were correlated to cancer immune escape and metastasis (76, 77). Increased *CCR1* activity was associated with CC chemokines such as *CCL3* and *CCL5*. Activation of *CCR1* in both cancer cells and myeloid cells is considered to promote cancer progression (78, 79). The increased expression of *KDR* and *FLT1* complex in cancer cells and macrophages was correlated with upregulated VEGFA signaling in cancer cells in solid areas, suggesting upregulated angiogenesis and vascular reconstitution (80). In summary, specific interaction in S areas reveals a session of a comprehensive process for cancer cells to decouple, hijack, and empower from TME.

Taken together, it could conceivably be hypothesized that the macrophage is one of the dominant factors driving the progression of the S components in mGGO by shaping the TME toward a tumor favoring hotbed since they constructed the majority of cell–cell interaction within the lesion, and many of the interacting pairs have been shown to be critical in tumor progression. The more complex immune surveillance environment in the S components could be generated by a diversified cancer cell population, which mostly originates from the GG components and branches into different subclusters when the mutation burden increases.

There are two previous single-cell sequence pieces of research studying mGGO (15, 16). Both of the studies have taken the whole mGGO as an entity. Tan’s study acclaimed a higher heterogeneity in solid LUAD than that in LUAD featured as GGO (15). Our study highlighted that a dominant cancer subcluster exists in GG areas, while S areas always consist of discrete subclusters. Wang’s study enrolled both pGGO and mGGO as a GGO group and compared it among solid LUAD and normal lungs. GGO is enriched with stress response programs (16). Our study further elaborates that the stress formation was polarized in different components of mGGO. However, our study was limited to the number of samples, we did a personalized analysis to increase the sensitivity of part of the outcomes, and a validation set would be better to enhance them. Taken together, our study decomposed intra-tumor heterogeneity of mGGO, revealing an alteration of cell components and transcriptomic features between the relative “pre-invasive” and “invasive” parts of the lesion and providing a possible developmental routine for it. This allows for a better knowledge of mGGO and the development of a novel management strategy for it.

## Data Availability Statement

The data presented is publicly accessible in the GEO database, accession number GSE203360.

## Ethics Statement

The studies involving human participants were reviewed and approved by the Ethics Committee Board of the Second Xiangya Hospital of Central South Hospital (2020084). The patients/participants provided their written informed consent to participate in this study.

## Author Contributions

(I) Conception and design: XC. (II) Administrative support: XC. (III) Screening and sampling: YuH, FY, YaH, BW, and QH. (IV) Collection and assembly of data: FY, YuH, YiT, and LW. (V) Data analysis and visualization: YuH and FY. (VI) Manuscript writing—original draft: YuH. (VII) Manuscript writing—review and editing: MP and XC. All authors listed have made a substantial, direct, and intellectual contribution to the work and approved it for publication.

## Funding

This work was supported by the National Natural Science Foundation of China (grant number 81972195, 82172879), the Hunan Provincial Key Area R&D Program (grant number 2019SK2253, 2021SK2020, 2021SK2013), the Natural Science Foundation of Hunan (2021JJ40871), the Scientific Research Program of Hunan Provincial Health Commission (grant number 20201047) and the Clinical Medical Technology Innovation Guide Project of Hunan Province (grant number 2020SK53408).

## Conflict of Interest

The authors declare that the research was conducted in the absence of any commercial or financial relationships that could be construed as a potential conflict of interest.

## Publisher’s Note

All claims expressed in this article are solely those of the authors and do not necessarily represent those of their affiliated organizations, or those of the publisher, the editors and the reviewers. Any product that may be evaluated in this article, or claim that may be made by its manufacturer, is not guaranteed or endorsed by the publisher.

## Supplementary Material

Supplementary Figure 1
**(A)** Synchronization of CT images and tissue appearances; **(B)** HE stains show the pathological margin of nlung (green arrow), GG component (red arrow), and S component (yellow arrow) within mGGO; **(C)** HE stains show different pathological appearances of disorganized fibrous necrosis (top left), third lymph nodes (top right), tumor cell infiltration (bottom left) and malignant acinus (bottom right) in S component; **(D)** Plot shows the wet weight of different components in mGGO per unit cells.

Supplementary Figure 2
**(A)** UMAP visualization shows sub-clustering of proliferating myeloid cells (left). CD1C and FCER1A were used to annotate DCs (top right). CD68 and MARCO were used to annotate macrophages (bottom right); **(B)** Proportion of major cell types within each sample; **(C)** IFA shows that macrophages (CD68, red) and cancer cells (NAPSA, green) are the dominant cell types in GG (top) and S (bottom) components, respectively. Scale bars:100 um; **(D)** UMAP visualization shows sub-clustering of annotated lymphocytes, mainly consist of CD8+ T cells and B cells.

Supplementary Figure 3
**(A)** The bubble plot shows upregulated signaling pathway of cancer cells in S component. KEGG pathway is on the left plot and Reactome pathway is on the right plot; **(B)** The bubble plot shows upregulated signaling pathway of cancer cells in GG component. KEGG pathway is on the left plot and Reactome pathway is on the right plot; **(C)** The single-cell entropy analysis reveals a slightly higher stemness of cancer cells in S components versus GG component.

Supplementary Figure 4
**(A)** The bubble plot shows significantly enriched KES Reactome pathways in Mac3, Mac6, Mac4, Mac5, respectively. The colors of bubbles represent the values of significance and the sizes represent the number of genes enriched in the pathway; **(B)** The bubble plot shows significantly enriched KEGG (left) and Reactome (right) pathways in Mac1; **(C)** Average expression of SPP1 in macrophage subclusters; **(D)** IFA shows enrichment of SPP^hi^ macrophages in S components (right) in comparison with the GG components (left). Yellow arrows indicate macrophages existing in the S components (right) featured as malignant acinus with upregulation of SPP1 (green). Red arrows indicate macrophages in the GG components (left) with poor expression level of SPP1. Scale bars:50 um.
