# Supplementary figures and images for: Single-Cell RNA Sequencing Unravels Distinct Tumor Microenvironment of Different Components of Lung Adenocarcinoma Featured as Mixed Ground-Glass Opacity
Source: Front Immunol. 2022 Jul 6;13:903513. doi: 10.3389/fimmu.2022.903513 (PMC9299373; doi:10.3389/fimmu.2022.903513)

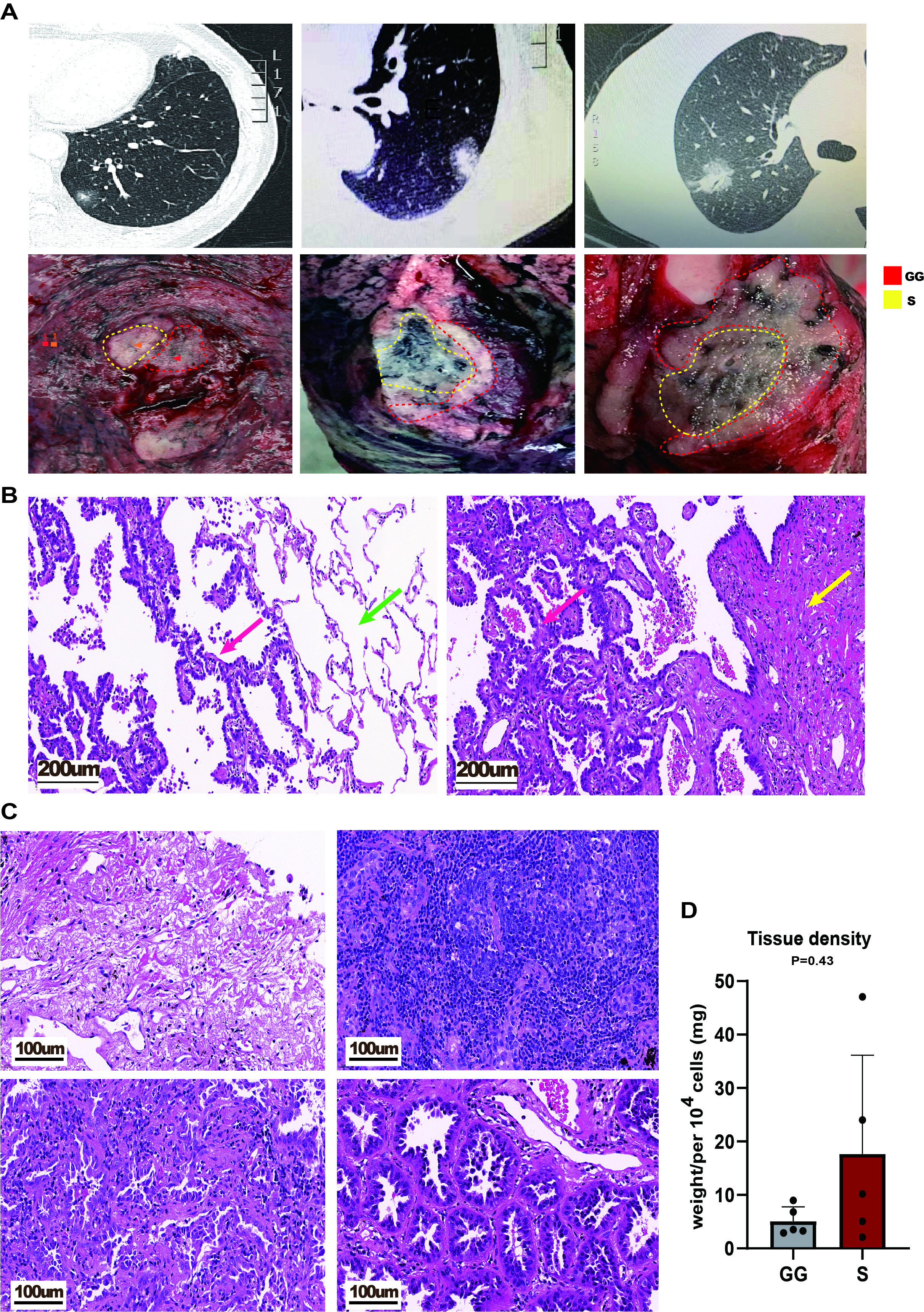

Supplement: Supplementary Figure 1 — (A) Synchronization of CT images and tissue appearances; (B) HE stains show the pathological margin of nlung (green arrow), GG component (red arrow), and S component (yellow arrow) within mGGO; (C) HE stains show different pathological appearances of disorganized fibrous necrosis (top left), third lymph nodes (top right), tumor cell infiltration (bottom left) and malignant acinus (bottom right) in S component; (D) Plot shows the wet weight of different components in mGGO per unit cells. [file Image_1.jpeg]

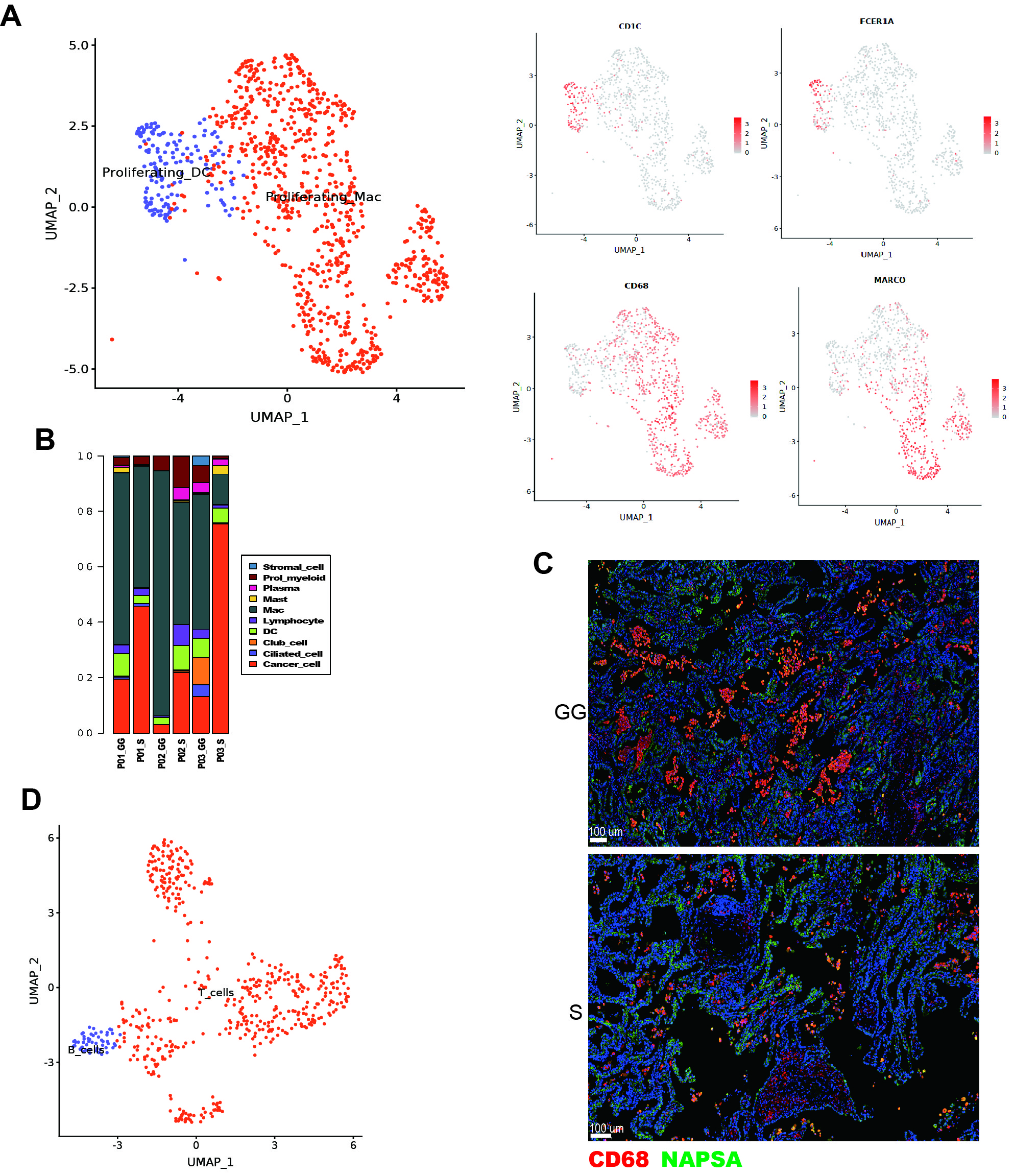

Supplement: Supplementary Figure 2 — (A) UMAP visualization shows sub-clustering of proliferating myeloid cells (left). CD1C and FCER1A were used to annotate DCs (top right). CD68 and MARCO were used to annotate macrophages (bottom right); (B) Proportion of major cell types within each sample; (C) IFA shows that macrophages (CD68, red) and cancer cells (NAPSA, green) are the dominant cell types in GG (top) and S (bottom) components, respectively. Scale bars:100 um; (D) UMAP visualization shows sub-clustering of annotated lymphocytes, mainly consist of CD8+ T cells and B cells. [file Image_2.jpeg]

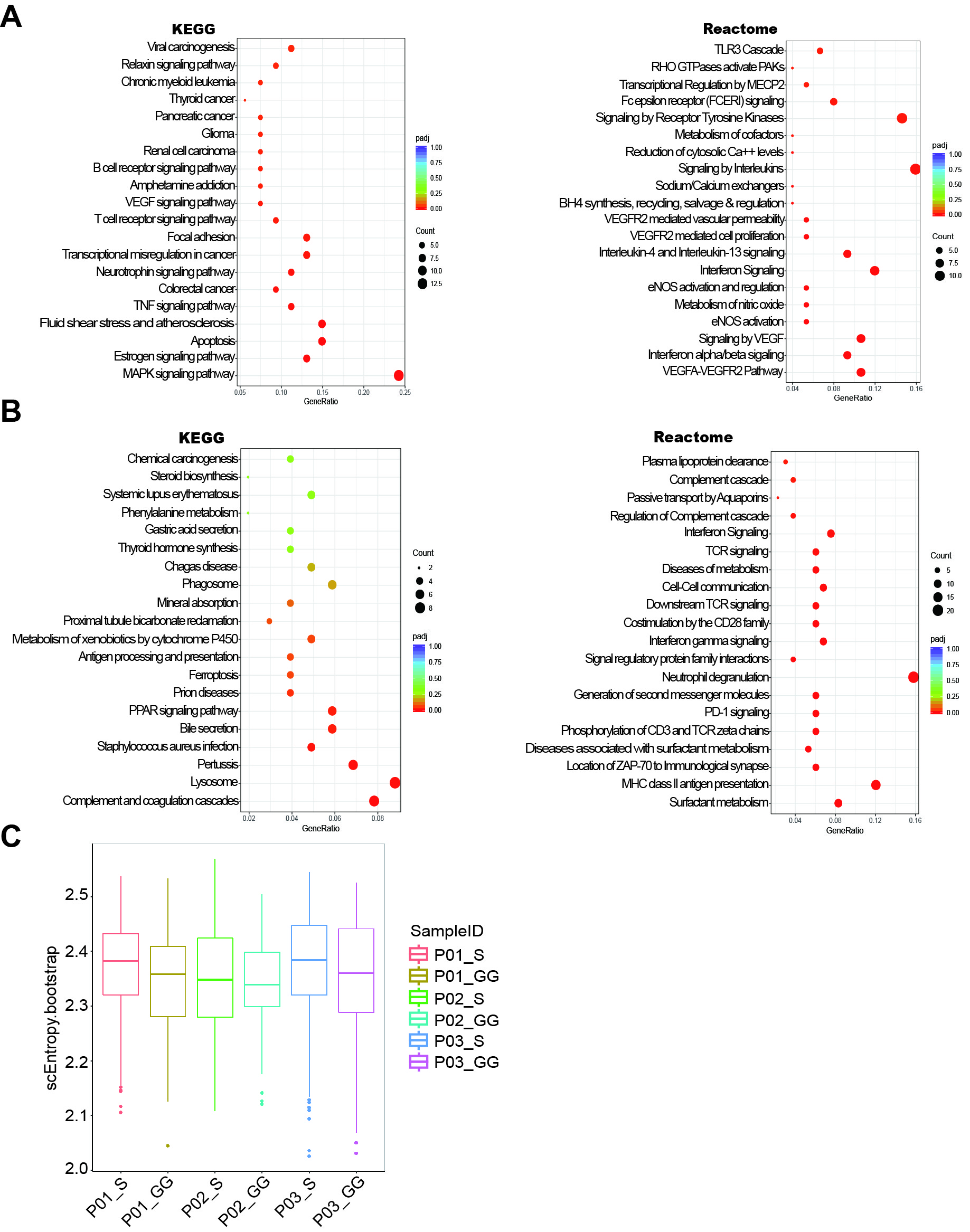

Supplement: Supplementary Figure 3 — (A) The bubble plot shows upregulated signaling pathway of cancer cells in S component. KEGG pathway is on the left plot and Reactome pathway is on the right plot; (B) The bubble plot shows upregulated signaling pathway of cancer cells in GG component. KEGG pathway is on the left plot and Reactome pathway is on the right plot; (C) The single-cell entropy analysis reveals a slightly higher stemness of cancer cells in S components versus GG component. [file Image_3.jpeg]

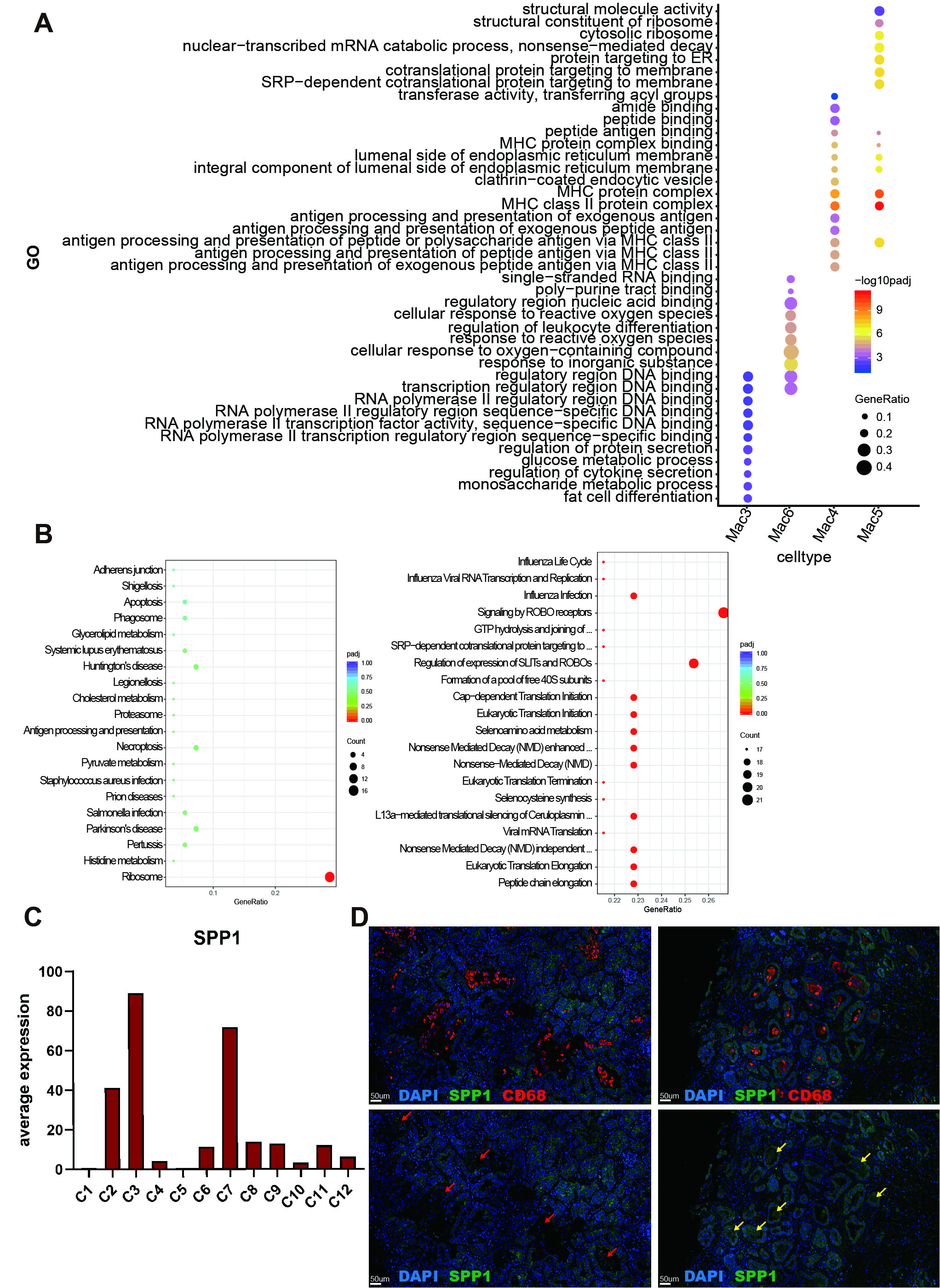

Supplement: Supplementary Figure 4 — (A) The bubble plot shows significantly enriched KES Reactome pathways in Mac3, Mac6, Mac4, Mac5, respectively. The colors of bubbles represent the values of significance and the sizes represent the number of genes enriched in the pathway; (B) The bubble plot shows significantly enriched KEGG (left) and Reactome (right) pathways in Mac1; (C) Average expression of SPP1 in macrophage subclusters; (D) IFA shows enrichment of SPPhi macrophages in S components (right) in comparison with the GG components (left). Yellow arrows indicate macrophages existing in the S components (right) featured as malignant acinus with upregulation of SPP1 (green). Red arrows indicate macrophages in the GG components (left) with poor expression level of SPP1. Scale bars:50 um. [file Image_4.jpeg]
