# Supplementary material for: Single-Cell RNA Sequencing Unravels Distinct Tumor Microenvironment of Different Components of Lung Adenocarcinoma Featured as Mixed Ground-Glass Opacity
Source: Front Immunol. 2022 Jul 6;13:903513. doi: 10.3389/fimmu.2022.903513 (PMC9299373; doi:10.3389/fimmu.2022.903513)
Supplement: Supplementary file 5 [file Table_1.docx]

**Supplementary table 1**

| Patients ID | Age/sex | diagnosis | Smoke history | test |
| --- | --- | --- | --- | --- |
| P01 | 53F | IAC | no | sc-seq, IHC |
| P02 | 66F | IAC | no | sc-seq, IHC |
| P03 | 74F | IAC | no | sc-seq, IHC |
| P04 | 36F | IAC | no | IHC, IF |
| P05 | 45F | IAC | no | IHC, IF |
| P06 | 54F | IAC | no | IHC, IF |
| P07 | 43F | IAC | no | IHC, IF |
| P08 | 58F | IAC | no | IHC, IF |
